# Novel thiazolone-benzenesulphonamide inhibitors of human and bacterial carbonic anhydrases

**DOI:** 10.1080/14756366.2022.2163243

**Published:** 2023-01-11

**Authors:** Morteza Abdoli, Viviana De Luca, Clemente Capasso, Claudiu T. Supuran, Raivis Žalubovskis

**Affiliations:** a Faculty of Materials Science and Applied Chemistry, Institute of Technology of Organic Chemistry, Riga Technical University, Riga, Latvia; b Department of Biology, Agriculture and Food Sciences, Institute of Biosciences and Bioresources, Napoli, Italy; c NEUROFARBA Department, Pharmaceutical and Nutraceutical Section, University of Florence, Florence, Italy; d Latvian Institute of Organic Synthesis, Riga, Latvia

**Keywords:** Carbonic anhydrase inhibitors, sulphonamides, thiazolones, *Mammaliicoccus (Staphylococcus) sciuri*, *Salmonella enterica* (*serovar Typhimurium*)

## Abstract

A small library of novel thiazolone-benzenesulphonamides has been prepared and evaluated for their ability to inhibit three human cytosolic carbonic anhydrases (hCA I, hCA II, and hCA VII) and three bacterial carbonic anhydrases (MscCAβ, StCA1, and StCA2). All investigated hCAs were inhibited by the prepared compounds **4a–4j** in the low nanomolar range. These compounds were effective hCA I inhibitors (K_I_s of 31.5–637.3 nM) and excellent hCA II (K_I_s in the range of 1.3–13.7 nM) and hCA VII inhibitors (K_I_s in the range of 0.9–14.6 nM). The most active analog in the series, 4-((4-oxo-5-propyl-4,5-dihydrothiazol-2-yl)amino)benzenesulphonamide **4d**, strongly inhibited bacterial MscCAβ, with K_I_ of 73.6 nM, considerably better than AAZ (K_I_ of 625 nM). The tested compounds displayed medium inhibitory potency against StCA1 (K_I_s of 69.2–163.3 nM) when compared to the standard drug (K_I_ of 59 nM). However, StCA2 was poorly inhibited by the sulphonamides reported here, with K_I_s in the micromolar range between 275.2 and 4875.0 nM.

## Introduction

**Figure 1.**
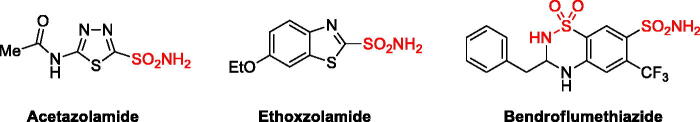
Selected example of FDA-approved sulphonamide carbonic anhydrase inhibitors.

The carbonic anhydrases (CAs; EC 4.2.1.1) are a ubiquitous family of metalloenzymes that are found in both prokaryotes and eukaryotes and catalyse the reversible reaction between carbon dioxide and water with formation of bicarbonate and a proton^1^. This simple reaction is fundamental to a variety of physiological and pathological processes in which cellular pH buffering plays an essential role^2^. In humans, 15 carbonic anhydrase isoforms (hCAs) have been identified till date, among which 3 have non-catalytic functions (VIII, X, and XI) and 12 are catalytically active (CA I–IV, VA–VB, VI–VII, IX, and XII–XIV)^3^. Emerging scientific evidence indicates that CAs are involved in an array of disorders, including haemolytic anaemia (CA I), edoema (CA II), altitude sickness (CA II), glaucoma (CA II, IV), obesity (CA VA), neuropathic pain (CA VII), cancer (CA IX, XII), sterility (CA XIII), and many others^4^. They are therefore a target for drug discovery and since so far dozens of CA inhibitors have been FDA-approved for the treatment/management of various diseases (see Figure 1)^5^. On the other hand, a possible new approach for the treatment of infectious diseases which are caused by microorganisms such as bacteria, fungi or parasites is based on the inhibition of carbonic anhydrases families being present in these organisms^6^.

Sulphonamide moiety (R-SO_2_NH_2_) is a common substructure in marketed small-molecule drugs^5^^,^^7^ and a relevant “warhead” to target CAs, mainly because of it strong zinc-binding affinity^8^. The large number of publications on the development of novel sulphonamide-based compounds and investigation of their inhibitory capacity against CAs for expanding the database of CAIs implies that the field is a hot topic in current drug discovery research^9^. However, a major challenge faced when designing hCA inhibitors for therapy is the lack of selectivity for a specific hCA isoform at least for the first and second generation compounds^10^.

For about two decades the tail approach was employed for developing isoform selective CA inhibitors. In this strategy, various moieties are attached to the terminal part of scaffolds of simple sulphonamides that may lead to interactions with the external part of the CA isoforms active site, which is the region at the entrance of the cavity that is more diverse than the active site residues between the 12 catalytically active human isoforms^11^.

**Figure 2.**
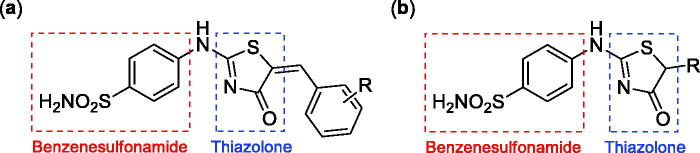
(a) General structure of thiazolone-benzenesulphonamides as selective hCA IX inhibitors developed by Hassan ^12^; (b) General structure of thiazolone-benzenesulphonamides discussed in the paper.

Very recently, the research group of Hassan has synthesised a small library of thiazolone-linked benzenesulphonamides (Figure 2(a)) and reported their strong and selective hCA IX inhibitory activity over hCA II, the most active human CA isoform^12^. Furthermore, it is reported that some of those compounds showed significant inhibitory effect against triple-negative breast cancer cell lines. In parallel with anticancer properties of thiazolone derivatives^13^, compounds possessing this heterocyclic ring have also been reported to exhibit antibacterial and antifungal activities^14^. Based on these literatures and in continuation of our interest in sulphonamide CAIs^15^, herein, we present a novel, highly efficient and step-economic method for the synthesis of thiazol-2(5H)-one-containing sulphanilamides (Figure 2(b)) and evaluate their capability to inhibit three bacterial and three human cytosolic carbonic anhydrases.

## Experimental section

### Chemistry

Reagents, starting materials and solvents were obtained from commercial sources and used as received. Thin-layer chromatography was performed on silica gel, spots were visualised with UV light (254 and 365 nm). NMR spectra were recorded on Bruker 500 spectrometer with chemical shifts values (δ) in ppm relative to TMS using the residual DMSO-d_6_ signal (^1^H 2.50; ^13^C 39.52). High-resolution mass spectra (HRMS) were recorded on a mass spectrometer with a Q-TOF micro mass analyser using the ESI technique.

### General procedure for the synthesis of compounds 4a–j

To a stirred solution of 1-(4-aminophenyl)thiourea (300 mg, 1.3 mmol) in DMF (4 ml) appropriate α-bromo ester (1.0 equiv., 1.3 mmol) was added at room temperature and the mixture was stirred at 25–40 °C for 15–48 h. Water (20. mL) was added to the reaction mixture and precipitate formed was collected by filtration, washed with water (100 ml) and air dried to afford the corresponding thiazolone-benzenesulphonamides (26–92%) as white solids.

#### 4-((4-Oxo-4,5-dihydrothiazol-2-yl)amino)benzenesulphonamide (4a)


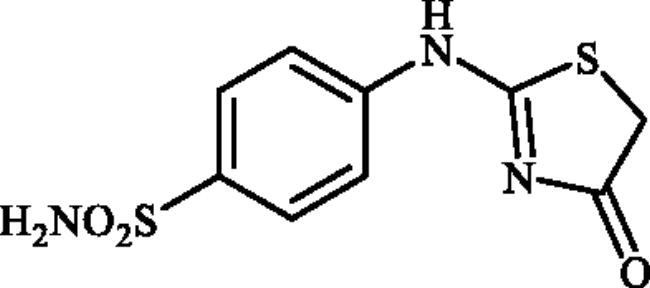
1-(4-Aminophenyl)thiourea (300 mg, 1.3 mmol) and ethyl 2-bromoacetate (0.143 ml, 1.3 mmol) were dissolved in DMF (4 ml). After stirring at room temperature for 15 h water (20 ml) was added and the precipitated product was filtered, washed with water (100 ml), and air dried to afford **4a** (322. mg, 92%) as a white powder.

^1^H NMR (500 MHz, DMSO-d_6_) *δ* = 4.06 (2H, s), 7.15 (1H, s), 7.35 (2H, s), 7.86 (3H, s), 11.49 and 11.93 (1H, s and s) ppm. ^13^C NMR (125 MHz, DMSO-d_6_) *δ* = 35.5, 39.4, 121.0, 122.4, 128.1, 140.6, 141.4, 151.9, 159.1, 175.3, 18.0.0, 189.3 ppm. HRMS (ESI) [M–H]^−^: *m*/*z* calcd for (C_9_H_8_N_3_O_3_S_2_) 270.0007. Found 270.0009.

#### 4-((5-Methyl-4-oxo-4,5-dihydrothiazol-2-yl)amino)benzenesulphonamide (4b)


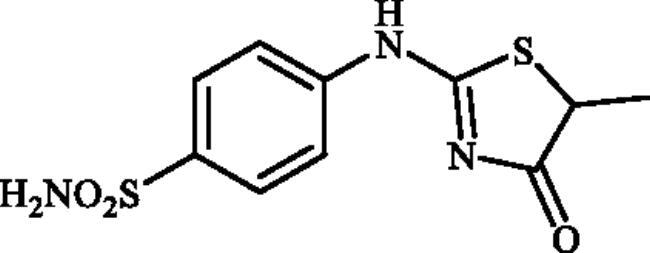
1-(4-Aminophenyl)thiourea (300 mg, 1.3 mmol) and ethyl 2-bromopropanoate (0.169 ml, 1.3 mmol) were dissolved in DMF (4 ml). After stirring at room temperature for 20 h water (20 ml) was added and the precipitated product was filtered, washed with water (100 ml), and air dried to afford **4b** (311 mg, 84%) as a white powder.

^1^H NMR (500 MHz, DMSO-d_6_) *δ* = 1.54 (3H, s), 4.37 (1H, s), 7.14 (1H, s), 7.35 (2H, s), 7.85 (3H, s), 11.48 and 11.93 (1H, s and s) ppm. ^13^C NMR (125 MHz, DMSO-d_6_) *δ* = 19.6, 45.0, 49.7, 121.1, 122.4, 128.1, 140.6, 142.3, 152.0, 157.2, 178.1, 178.5, 192.0 ppm. HRMS (ESI) [M–H]^−^: *m*/*z* calcd for (C_10_H_10_N_3_O_3_S_2_) 284.0164. Found 284.0166.

#### 4-((5-Ethyl-4-oxo-4,5-dihydrothiazol-2-yl)amino)benzenesulphonamide (4c)


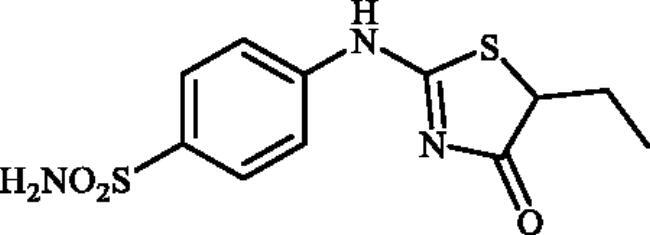
1-(4-Aminophenyl)thiourea (300 mg, 1.3 mmol) and ethyl 2-bromobutanoate (0.191 ml, 1.3 mmol) were dissolved in DMF (4 ml). After stirring at room temperature for 20 h water (20 ml) was added and the precipitated product was filtered, washed with water (100 ml), and air dried to afford **4c** (298 mg, 77%) as a white powder.

^1^H NMR (500 MHz, DMSO-d_6_) *δ* = 0.95 (3H, s), 1.85 (1H, s), 2.00 (1H, s), 4.41 (1H, s), 7.15 (1H, s), 7.35 (2H, s), 7.86 (3H, s), 11.51 and 11.94 (1H, s and s) ppm. ^13^C NMR (125 MHz, DMSO-d_6_) *δ* = 11.4, 11.9, 26.4, 52.1, 57.1, 121.1, 12.4, 140.6, 142.3, 152.1, 157.3, 177.2, 178.7, 191.0 ppm. HRMS (ESI) [M–H]^−^: *m*/*z* calcd for (C_11_H_12_N_3_O_3_S_2_) 298.0320. Found 298.0327.

#### 4-((4-Oxo-5-propyl-4,5-dihydrothiazol-2-yl)amino)benzenesulphonamide (4d)


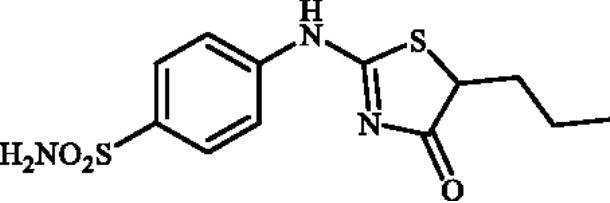
1-(4-Aminophenyl)thiourea (300 mg, 1.3 mmol) and ethyl 2-bromopentanoate (0.426 ml, 2.6 mmol) were dissolved in DMF (4 ml). After stirring at room temperature for 36 h water (20 ml) was added and the precipitated product was filtered, washed with water (100 ml), and air dried to afford **4d** (264 mg, 65%) as a white powder.

^1^H NMR (500 MHz, DMSO-d_6_) *δ* = 0.91 (3H, s), 1.33 (1H, s), 1.44 (1H, s), 1.77 (1H, s), 1.99 (1H, s), 4.41 (1H, s), 7.15 (1H, s), 7.34 (2H, s), 7.86 (3H, s), 11.49 and 11.95 (1H, s and s) ppm. ^13^C NMR (125 MHz, DMSO-d_6_) *δ* = 14.4, 20.6, 21.4, 35.4, 35.6, 50.7, 55.7, 121.1, 122.4, 127.9, 128.1, 140.7, 142.4, 152.1, 157.3, 177.4, 178.7, 191.2 ppm. HRMS (ESI) [M–H]^−^: *m*/*z* calcd for (C_12_H_14_N_3_O_3_S_2_) 312.0477. Found 312.0478.

#### 4-((5-Isopropyl-4-oxo-4,5-dihydrothiazol-2-yl)amino)benzenesulphonamide (4e)


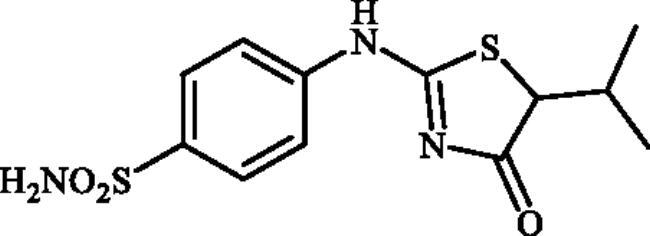
1-(4-Aminophenyl)thiourea (300 mg, 1.3 mmol) and ethyl 2-bromo-3-methylbutanoate (0.424 ml, 2.6 mmol) were dissolved in DMF (4 ml). After stirring at 40 °C for 36 h water (20 ml) was added and the precipitated product was filtered, washed with water (100 ml), and air dried to afford **4e** (106 mg, 26%) as a white powder.

^1^H NMR (500 MHz, DMSO-d_6_) *δ* = 0.94 (5H, d, *J* = 9.2 Hz), 1.05 (1H, s), 2.42 (1H, s), 4.50 (1H, s), 7.16 (1H, s), 7.34 (2H, s), 7.86 (3H, s), 11.52 and 11.97 (1H, s and s) ppm. ^13^C NMR (125 MHz, DMSO-d_6_) *δ* = 16.8, 20.8, 22.2, 30.4, 57.2, 62.6, 120.6, 121.9, 127.4, 127.6, 140.1, 141.8, 151.6, 156.7, 176.2, 178.3, 190.0 ppm. HRMS (ESI) [M–H]^−^: *m*/*z* calcd for (C_12_H_14_N_3_O_3_S_2_) 312.0477. Found 312.0475.

#### 4-((5-Butyl-4-oxo-4,5-dihydrothiazol-2-yl)amino)benzenesulphonamide (4f)


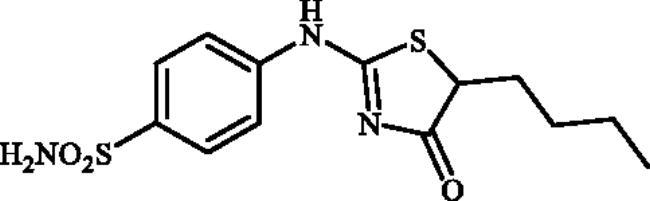
1-(4-Aminophenyl)thiourea (300 mg, 1.3 mmol) and ethyl 2-bromohexanoate (0.23.7 ml, 1.3 mmol) were dissolved in DMF (4 ml). After stirring at room temperature for 36 h water (20 ml) was added and the precipitated product was filtered, washed with water (100 ml), and air dried to afford **4f** (281 mg, 66%) as a white powder.

^1^H NMR (500 MHz, DMSO-d_6_) *δ* = 0.60 (3H, s), 1.03 (4H, s), 1.14 (1H, s), 1.50 (1H, s), 1.74 (1H, s), 4.13 (1H, s), 6.87 (1H, s), 7.06 (2H, s), 7.57 (3H, s), 11.21 and 11.67 (1H, s and s) ppm. ^13^C NMR (125 MHz, DMSO-d_6_) *δ* = 14.7, 22.6, 29.3, 30.1, 32.98, 50.8, 55.9, 121.1, 122.4, 127.9, 128.1, 140.7, 142.3, 152.1, 157.2, 177.3, 178.7, 191.2 ppm. HRMS (ESI) [M–H]^−^: *m*/*z* calcd for (C_13_H_16_N_3_O_3_S_2_) 326.0633. Found 326.0634.

#### 4-((5-Hexyl-4-oxo-4,5-dihydrothiazol-2-yl)amino)benzenesulphonamide (4g)


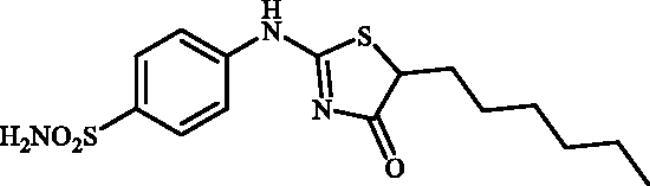
1-(4-Aminophenyl)thiourea (300 mg, 1.3 mmol) and ethyl 2-bromooctanoate (0.546 ml, 2.6 mmol) were dissolved in DMF (4 ml). After stirring at room temperature for 48 h water (20 ml) was added and the precipitated product was filtered, washed with water (100 ml), and air dried to afford **4g** (221 mg, 48%) as a white powder.

^1^H NMR (500 MHz, DMSO-d_6_) *δ =*  0.87 (3H, s), 1.27 (6H, s), 1.42 (2H, s), 1.77 (1H, s), 2.02 (1H, s), 7.14 (1H, s), 7.34 (2H, s), 7.85 (3H, s), 11.49 and 11.81 (1H, s and s) ppm. ^13^C NMR (125 MHz, DMSO-d_6_) *δ* = 14.8, 22.9, 27.1, 27.8, 29.1, 31.9, 33.2, 50.8, 55.8, 121.0, 122.3, 127.9, 128.1, 140.6, 142.3, 152.0, 157.3, 177.3, 178.5, 191.1 ppm. HRMS (ESI) [M–H]^−^: *m*/*z* calcd for (C_15_H_20_N_3_O_3_S_2_) 354.0946. Found 354.0955.

#### 4-((4-Oxo-5-phenyl-4,5-dihydrothiazol-2-yl)amino)benzenesulphonamide (4h)


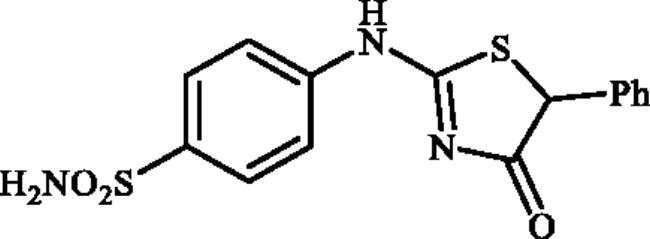
1-(4-Aminophenyl)thiourea (300 mg, 1.3 mmol) and ethyl 2-bromo-2-phenylacetate (0.546 ml, 1.3 mmol) were dissolved in DMF (4 ml). After stirring at room temperature for 20 h water (20 ml) was added and the precipitated product was filtered, washed with water (100 ml), and air dried to afford 4 h (397 mg, 88%) as a white powder.

^1^H NMR (500 MHz, DMSO-d_6_) *δ* = 0.60 (3H, s), 5.65 (1H, d, *J* = 6.2 Hz), 7.25 (1H, s), 7.41 (7H, d, *J* = 6.2 Hz), 7.88–7.98 (3H, m), 11.71 and 12.23 (1H, s and s) ppm. ^13^C NMR (125 MHz, DMSO-d_6_) *δ* = 53.9, 58.5, 121.3, 122.5, 127.9, 128.2, 129.1, 129.4, 129.9, 137.2, 137.7, 140.9, 142.3, 151.5, 157.5, 176.2, 178.7, 189.4 ppm. HRMS (ESI) [M–H]^−^: *m*/*z* calcd for (C_15_H_12_N_3_O_3_S_2_) 346.0320. Found 346.0322.

#### 4-((5-(2-Bromoethyl)-4-oxo-4,5-dihydrothiazol-2-yl)amino)benzenesulphonamide (4i)


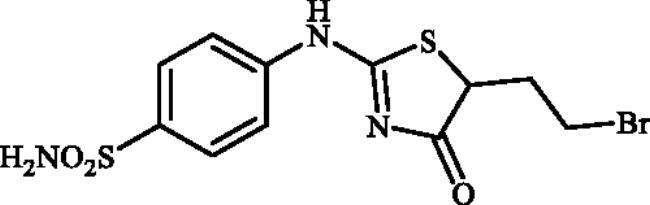
1-(4-Aminophenyl)thiourea (300 mg, 1.3 mmol) and ethyl 2,4-dibromobutanoate (0.205 ml, 1.3 mmol) were dissolved in DMF (4 ml). After stirring at room temperature for 20 h water (20 ml) was added and the precipitated product was filtered, washed with water (100 ml), and air dried to afford **4i** (396 mg, 81%) as a white powder.

^1^H NMR (500 MHz, DMSO-d_6_) *δ* = 2.32 (1H, s), 2.62 (1H, s), 3.67 (2H, s), 4.48 (1H, s), 7.16 (1H, s), 7.35 (2H, s), 7.86 (3H, s), 11.58 and 12.00 (1H, s and s) ppm. ^13^C NMR (125 MHz, DMSO-d_6_) *δ* = 32.6, 33.5, 36.4, 36.9, 49.5, 54.3, 121.2, 122.4, 127.9, 128.1, 140.7, 142.3, 151.8, 157.3, 176.9, 178.7, 190.4 ppm. HRMS (ESI) [M–H]^−^: *m*/*z* calcd for (C_11_H_11_N_3_O_3_S_2_Br) 375.9425. Found 377.9416.

#### 4-((5-Fluoro-4-oxo-4,5-dihydrothiazol-2-yl)amino)benzenesulphonamide (4j)


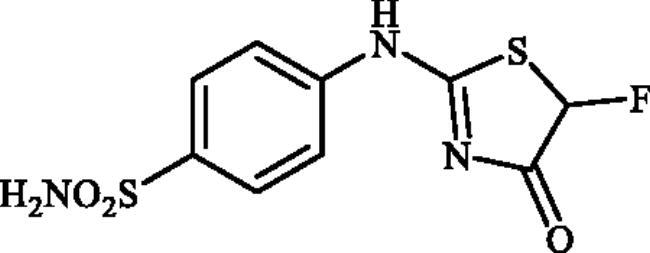
1-(4-Aminophenyl)thiourea (300 mg, 1.3 mmol) and ethyl 2-bromo-2-fluoroacetate (0.153 ml, 1.3 mmol) were dissolved in DMF (4 ml). After stirring at room temperature for 20 h the reaction mixture was transferred to a separating funnel and mixture of water/dichloromethane (15:10 ml) was added and it was shaken vigorously for a few minutes. After 1 h, the precipitated product was filtered, washed with water (100 ml) and CH_2_Cl_2_ (20 ml), and air dried to afford **4j** (247 mg, 66%) as a white powder.

^1^H NMR (500 MHz, DMSO-d_6_) *δ* = 6.76 (1H, d, *J* = 44.6 Hz), 7.18 (1H, d, *J* = 5.0 Hz), 7.40 (2H, d, *J* = 14.2 Hz), 7.88 (1H, d, *J* = 5.4 Hz), 7.92 (2H, s), 11.97 and 12.61 (1H, s and s) ppm. ^13^C NMR (125 MHz, DMSO-d_6_) *δ* = 94.0 (d, *J* = 221.8 Hz), 97.6 (d, *J* = 223.5 Hz), 122.2 (d, *J* = 42.0 Hz), 128.1 (d, *J* = 33.8 Hz), 141.4, 141.6 (d, *J* = 9.8 Hz), 150.9, 153.9, 171.0, 176.1, 184.6 (d, *J* = 13.6 Hz) ppm. HRMS (ESI) [M–H]^−^: *m*/*z* calcd for (C_9_H_7_N_3_O_3_FS_2_) 287.9913. Found 287.9910.

## CA inhibition assay

An applied photophysics stopped-flow instrument has been used for assaying the CA catalysed CO_2_ hydration activity^16^. Phenol red (at a concentration of 0.2 mM) was used as indicator, working at the absorbance maximum of 557 nm, with 20 mM Hepes (pH 7.5) as buffer for α-CAs or 20 mM TRIS (pH 8.4) as buffer for β-CAs, and 20 mM Na_2_SO_4_ (for maintaining constant the ionic strength), following the initial rates of the CA-catalysed CO_2_ hydration reaction for a period of 10 – 100 s. The CO_2_ concentrations ranged from 1.7 to 17 mM for the determination of the kinetic parameters and inhibition constants. For each inhibitor, at least six traces of the initial 5 – 10% of the reaction have been used for determining the initial velocity. The uncatalysed rates were determined in the same manner and subtracted from the total observed rates. Stock solutions of inhibitor (0.1 mM) were prepared in distilled – deionised water, and dilutions up to 0.1 nM were done thereafter with the assay buffer. Inhibitor and enzyme solutions were preincubated together for 15 min at room temperature prior to assay in order to allow for the formation of the enzyme – inhibitor complex. The inhibition constants were obtained by nonlinear least-squares methods using PRISM 3 and the Cheng–Prusoff equation, as reported earlier^17–19^, and represent the mean from at least three different determinations. All CA isoforms were recombinant ones obtained in-house as reported earlier^20–22^ and their concentrations in the assay system ranged between 10 and 14 nM.

## Results and discussion

### Chemistry

**Scheme 1.**
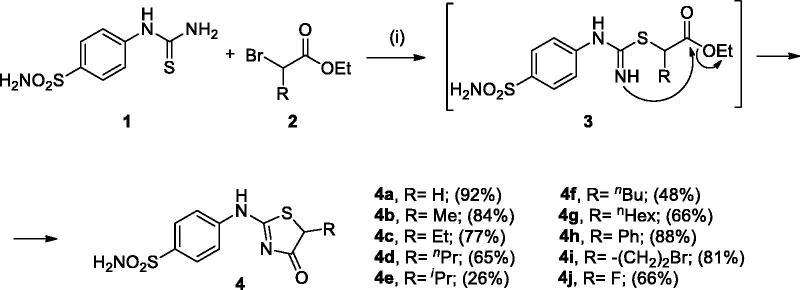
Reagents and conditions: (i) DMF, 25–40 °C, 15–48 h.

**Figure 3.**
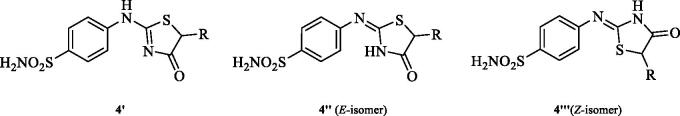
Representative tautomers of compounds **4**.

In Scheme 1, the synthesis of the target compounds reported here. The synthesis starts with the selective catalyst- and additive-free *S*-alkylation of easily accessible 4-thioureidobenzenesulphonamide **1** with commercially available α-bromo esters **2** under mild conditions. Following spontaneous intramolecular cyclisation in *in situ* generated ethyl 2-((*N*-(4-sulphamoylphenyl)carbamimidoyl)thio)acetate intermediate **3** into the desired products **4** in fair to excellent yields, ranging from 26% to 92%. The analytical and spectroscopic data (NMR and MS spectra) of the prepared compounds are in agreement with the proposed structures. Noteworthy, as observed in NMRs all compounds **4** exist as a mixture of tautomers (Figure 3)^23^. All synthesised inhibitors are novel and not known in literature.

### Carbonic anhydrase inhibition

The newly synthesised target compounds **4a-4j** were evaluated for their inhibitory activity towards three human (h) CA isoforms, the cytosolic hCA I, II, and VII, as well as three bacterial β-CAs from one gram-positive bacteria, *Mammaliicoccus (Staphylococcus) sciuri*, MscCAβ, and one gram-negative bacteria, *Salmonella enterica (serovar Typhimurium),* StCA1 and StCA2, by using a stopped-flow CO_2_ hydrase assay^16^.

Some important information from the data of Table 1, where the inhibition data of these enzymes are presented, are listed below:

**Table 1.** Inhibition data of human CA isoforms hCA I, II, and VII and bacterial β-CA isoforms MscCA, from *Mammaliicoccus (Staphylococcus) sciuri*, and StCA1 and StCA2, from *Salmonella enterica* (serovar *Typhimurium*) with compounds **4a–j** in comparison with the standard sulphonamide inhibitor **AAZ** by a stopped flow CO_2_ hydrase assay.

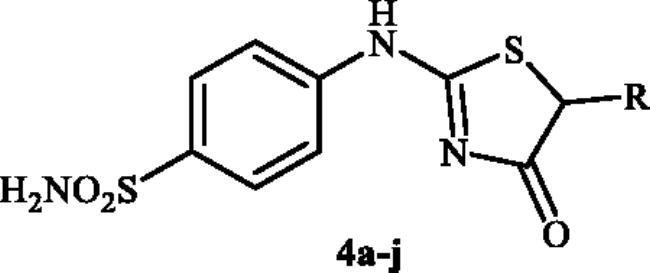							
Compound	R	K_I_ (nM)^a^					
		hCA I	hCA II	hCA VII	MscCAβ	StCA1	StCA2
**4a**	–H	63.9	3.4	1.4	786.6	82.4	275.2
**4b**	–CH_3_	31.5	4.3	0.9	481.4	89.9	538.1
**4c**	–CH_2_CH_3_	66.6	3.8	1.2	411.0	86.1	480.8
**4d**	–(CH_2_)_2_CH_3_	70.7	2.3	2.0	73.6	83.3	913.1
**4e**	–CH(CH_3_)_2_	85.4	7.3	7.5	701.3	81.1	866.9
**4f**	–(CH_2_)_3_CH_3_	75.0	3.2	6.2	2425.0	72.4	904.0
**4g**	–(CH_2_)_5_CH_3_	637.3	13.7	14.6	4026.3	83.6	4875.0
**4h**	–C_6_H_5_	90.4	1.3	7.3	1056.7	163.3	797.4
**4i**	–(CH_2_)_2_Br	74.6	2.2	2.1	924.5	69.2	515.1
**4j**	–F	137.5	3.7	1.9	848.7	90.9	955.9
**AAZ**	–	250	12.5	2.5	625	59	84

^a^Mean from three different assays, by a stopped flow technique (errors were in the range of ±5–10% of the reported values).

- Ninety percent of the evaluated compounds exhibited superior potency against the slowest cytosolic human isoform hCA I with K_Is_ in the range of 31.5–137.5 nM compared to the standard drug (acetazolamide; AAZ) whose K_I_ value is 250 nM. The SAR analysis showed that the inhibitory activity was decreased with the increased chain length in alkyl-substituted compounds **4b–4g**. Therefore, the best inhibitor was compound **4b** (K_I_ of 31.5 nM) with the smallest alkyl group (Me), whereas the poorest inhibitor was compound **4g** (K_I_ of 637.3 nM) with the longest alkyl group (*n*-Hex). However, the replacement of the aliphatic groups with a hydrogen atom did not increase the inhibitory activity as unsubstituted compound **4a** showed almost equal inhibitory activity with Et-substituted one **4c**. Additionally, no remarkable change was observed when the aliphatic groups were replaced with a benzene ring so that Ph-substituted compound **4h** displayed almost the same inhibitory activity with *^i^*Pr-substituted one **4e**. However, the inhibitory activity was decreased with the insertion of a fluorine atom at the C5-position. In summary, the relative inhibitory rates of newly designed compounds against hCA I followed the order: Methyl (C1) substituted- > unsubstituted- ≈ medium aliphatic chain (C2-C4) substituted- ≈ benzene-substituted ≥ F-substituted- > long aliphatic chain (C6) substituted derivatives.
- Against the fastest cytosolic human isoform hCA II, similar to hCA I, except long aliphatic chain (C6)-substituted thiazolone-benzenesulphonamide **4g** with K_I_ values of 13.7 nM, other compounds acted as more effective inhibitors (K_I_s in the range of 1.3–7.3 nM) compared to the standard drug (K_I_ of 12.5 nM). Therefore, all substitution patterns present in these compounds lead to highly effective CAIs. It should be mentioned that the only aryl-substituted derivative **4h** of the series exhibited the best inhibition for this isozyme which was 10-fold more effective than AAZ. Thus, synthesis and evaluation of novel aryl-substituted thiazolone-benzenesulphonamide may be useful for the development of effective and selective inhibitor of hCA II.
- The brain-associated cytosolic isoform hCA VII which was recently validated as therapeutic target for the relief of neuropathic pain^24^ showed a rather similar inhibition profile with hCA I, with sulphonamides investigated here. Therefore, methyl-substituted compound **4b** was the strongest inhibitor with a K_I_ of 0.9 nM and *^n^*Hex-substituted compound **4g**, showed poorest inhibitory capacities with a K_I_ of 14.6 nM. It is worth mentioning that 60% of the compounds investigated here (**4a–d, 4i**, **4j**), showed even better inhibitory activities against hCA VII in comparison with the reference drug.
- The bacterial β-CA from *Mammaliicoccus (Staphylococcus) sciuri*, MscCAβ, was effectively inhibited by some of the obtained sulphonamides, with the K_I_ values in the range of 73.6 and 4026.3 nM. Indeed, Among the 10 investigated compounds, three exhibited superior inhibitory activity compared to AAZ which all are alkyl-substituted derivatives. Compound **4d** showed the best inhibition against MscCA with a K_I_ of 73.6 nM, which was 8-fold higher than that of AAZ (K_I_ of 625 nM). Although this compound **4d** displayed negligible selectivity for MscCA over StCA1 (MscCA/StCA1:1.4), its high selectivity index greater than 10 for MscCAversus StCA2 is remarkable.
- One of the *S. enterica (*serovar *Typhimurium)* CA isoform, StCA1, was effectively inhibited by the sulphonamides reported here, with K_I_s in the range between 69.2 and 163.3 nM. The most promising compound, **4g**, showed the most significant selectivity for this isozyme versus MscCAβ and StCA2, 48- and 58-fold higher selectivity, respectively. Therefore, this compound is an excellent candidate for future *in vivo* preclinical studies.
- The second *S. enterica (*serovar *Typhimurium)* CA isoform, StCA2, was on the other hand poorly inhibited with targeted sulphonamides compared to MscCAβ and StCA1, and the K_Is_ were in the range of 275.2–4875.0 nM. Among the tested compounds, unsubstituted derivative **4a** demonstrated the most potent StCA2 inhibitory activity with a K_I_ of 275.2 nM, although the activity was three-fold lower than the reference compound (K_I_ of 84 nM).

## Conclusion

A small library of C5-functionalized thiazol-2(5H)-one-containing sulphonamide derivatives **4a–4j** has been prepared through a high atom- and step-economical reaction between easily accessible 4-thioureidobenzenesulphonamide and commercially available α-bromo esters under mild and additive-free conditions. These compounds were tested for their inhibition of three cytosolic human carbonic anhydrase isoforms (hCA I, hCA II, and hCA VII) as well as three bacterial enzymes belonging to the β-CA class (MscCAβ, StCA1, and StCA2). The results showed that majority of the examined compounds effectively inhibited hCAs, even better than the standard drug (AAZ). Noteworthy, in all cases these compounds showed significant selectivity for hCA II and hCA VII over hCA I. However, they did not show selectivity for the inhibition of hCA II versus hCA VII. The bacterial CAs were also inhibited by these compounds, but not as effectively as the hCAs. Importantly, compound **4g** which showed the poorest capacity to inhibit human CAs, inhibited StCA1 almost in the same range of AAZ, while it has a 48- and 58-fold high selectivity for this isozyme versus MscCA and StCA2, respectively. The activity of this analog against StCA1 is especially noteworthy, and suggests that it is an excellent candidate for future *in vivo* preclinical studies.
